# Invasive *Dreissena* Mussel Coastal Transport From an Already Invaded Estuary to a Nearby Archipelago Detected in DNA and Zooplankton Surveys

**DOI:** 10.3389/fmars.2022.818738

**Published:** 2022-02-21

**Authors:** Courtney E. Larson, Jonathan T. Barge, Chelsea L. Hatzenbuhler, Joel C. Hoffman, Greg S. Peterson, Erik M. Pilgrim, Barry Wiechman, Christopher B. Rees, Anett S. Trebitz

**Affiliations:** 1 U.S. Environmental Protection Agency, Office of Research and Development, Duluth, MN, United States,; 2 Department of Biology, University of Minnesota Duluth, Duluth, MN, United States,; 3 Oak Ridge Institute for Science and Education, Oak Ridge, TN, United States,; 4 SpecPro Professional Services, Duluth, MN, United States,; 5 U.S. Environmental Protection Agency, Office of Research and Development, Cincinnati, OH, United States,; 6 Aptim Federal Services, LLC, Baton Rouge, LA, United States,; 7 U.S. Fish and Wildlife Service, Northeast Fishery Center, Lamar, PA, United States

**Keywords:** *Dreissena*, Lake Superior, current transport, eDNA, qPCR

## Abstract

Coastal waters of Lake Superior are generally inhospitable to the establishment of invasive *Dreissena spp*. mussels (both *Dreissena polymorpha* and *Dreissena bugensis*). *Dreissena* have inhabited the Saint Louis River estuary (SLRE; largest commercial port in the Laurentian Great Lakes) for over three decades, but only in the last few years have small colonies been found in the Apostle Islands National Lakeshore (APIS, an archipelago situated 85 km to the east of SLRE) A 2017 survey determined a low abundance *Dreissena* spatial distribution in APIS, with the largest colonies on the north and west islands which suggested potential veliger transport from the SLRE *via* longshore currents. Our objective in this study was to determine if *Dreissena* veligers are transported by currents at low densities along the south shore of Lake Superior from the SLRE to APIS. To do so, we used both eDNA (water and passive substrate samples) and zooplankton collection methods at eight sites evenly spaced between the SLRE and APIS with three sampling times over five weeks. *Dreissena* veligers were consistently detected along the south shore, although at low abundances (veligers per m^3^ range = 0–690, median = 8), and for every 1 km increase in distance from the SLRE, both veliger counts and water eDNA copy numbers decreased on average by 5 and 7%, respectively. *D. polymorpha* (suited to estuary habitats) was detected two times more than *D. bugensis* (better suited to deep-lake habitats). There was not a trend in the veliger size distribution along the south shore, and temperature and calcium concentrations fluctuated around the threshold for *Dreissena* veliger and adult development, averaging 11.0°C and 14.8 ppm, respectively. Three zooplankton taxa representative of the estuary community–*Daphnia retrocurva*, *Diaphanosoma birgei*, and *Mesocyclops* copepodites–decreased as the distance from the SLRE increased mirroring *Dreissena* veliger abundance patterns. Findings represent multiple sources of evidence of a propagule “conveyor belt” for *Dreissena* along the south shore of Lake Superior. We conclude that veligers are functioning as a propagule, using coastal currents to spread from the point of invasion, thereby traversing coastal habitat previously reported as inhospitable to distant habitats suitable for colonization.

## INTRODUCTION

Currents can drive larval transport of invasive species to new habitats. Many aquatic benthic organisms have a two-part life history with a sedentary adult phase and a drifting larval phase. This larval phase disperses *via* currents, which leads to invasion “source-sink” processes (Pineda et al., 2007). Long-distance dispersal may occur when physical conditions allow drift across long distances to new suitable habitats (Hastings et al., 2005; Kinlan et al., 2005). Marine benthic invertebrate dispersal can range from tens to hundreds of kilometers (Kinlan and Gaines, 2003).

Environmental characteristics can limit or expand the dispersal of invasive invertebrate larvae. For example, surface currents drove dispersal through unsuitable to suitable environments of the invasive jellyfish *Phyllorhiza punctata* in the Gulf of Mexico (Johnson et al., 2005), red king crab *Paralithodes camtschaticus* larvae in Norway’s northern coast (Pedersen et al., 2006), and the Chinese mitten crab *Eriocheir sinensis* in the Delaware Bay (Tilburg et al., 2011). Additionally, the European green crab *Carcinus maenas* was transported by currents across the west coast of North America bridging temporal and temperature limitations (See and Feist, 2010). The furthest north expansion of this invasive species occurred during the fall of an El Niño year, demonstrating temporal variability in the spread, and current velocity had a two times greater effect on transport than temperature (See and Feist, 2010).

The Laurentian Great Lakes (hereafter, Great Lakes) contain freshwater coastal habitat with differences and similarities to traditional oceanic coastal habitat that make an interesting basis of comparison for larval transport. Although a salinity gradient is not present in Great Lakes estuarine habitat, the mesophysical processes of upwelling and currents are comparable to oceans (Bedford, 1992), and the scale of coastal habitat is similarly large, with the Great Lakes containing as much coastline length as the Atlantic seaboard (Young and Janssen, 2009). The coastal habitat of oceans and the Great Lakes are considered a hot spot for aquatic invasive invertebrates, with many similar anthropological vectors, such as large ship traffic and recreational boating (Ricciardi, 2006; O’Malia et al., 2018). Yet in the Great Lakes, there are not nearly as many taxa that have pelagic early life stages compared to oceans, so current-driven transport has been less explored. One Great Lakes case study predicted larval transport from major harbors in lakes Michigan and Erie for potential invader golden mussels using a three-dimensional particle transport model (Beletsky et al., 2017). It predicted that in 2 weeks of drift time mussels can travel over 200 km, and larval advection may be important for aquatic invasive species (AIS) dispersal in the Great Lakes.

*Dreissena* is a genus of mussels containing *Dreissena polymorpha* (zebra mussel) and *Dreissena bugensis* (quagga mussel), both of which are native to eastern Europe and western Asia and invasive in the Laurentian Great Lakes (Nalepa and Schloesser, 2013; U.S. Geological Survey, 2020). Adult *Dreissena* colonize benthic structures and filter feed, and immature veligers disperse in the zooplankton community, with *D. polymorpha* and *D. bugensis* occupying similar ecological niches (Karatayev et al., 2015), although *D. bugensis* tends to occur in deeper and colder waters and is better able to colonize soft sediments. *D. polymorpha* first invaded Lake Erie in 1986, spreading to all the Great Lakes by 1990. After *D. bugensis* invaded in 1989, it spread across the great lakes, overtaking *D. polymorpha* populations as the dominant mussel in both deep and soft substrate habitats. There is a wide range of negative effects reported from Great Lakes *Dreissena* populations, including alterations to food webs, community structure, water clarity, and nutrient availability, making these invasions a significant conservation concern over the past >30 years (Higgins and Zanden, 2010; Karatayev et al., 2015). Most published studies on *Dreissena* impacts in the Great Lakes focus on the bottom-settled stages, but veliger abundances are seasonally high enough in the lower Great Lakes that impacts on zooplankton are also suspected (Bowen et al., 2018).

In contrast to the other four Laurentian Great Lakes – whose bottoms are blanketed with *Dreissena* – Lake Superior presently has only a few spatially limited *Dreissena* colonies in the lake proper, which is thought to be because of its inhospitable environmental conditions. One factor limiting *Dreissena* colonization may be lower calcium (Ca^++^) concentrations in Lake Superior compared to other great lakes, which limits shell growth (Lafrancois et al., 2018). Additionally, Lake Superior is colder (in the summer) and more oligotrophic than the other great lakes, and *Dreissena* is limited by the low productivity and temperature (Grigorovich et al., 2003). Yet, small and scattered *Dreissena* colonies have been recorded in recent years at several locations around the Apostle Islands National Lakeshore (APIS). The one established *Dreissena* population is in the barrier-beach protected Saint Louis River estuary (SLRE; also known as the Duluth-Superior harbor), which is located at the far western end of Lake Superior and has had *Dreissena* present since 1989 (Trebitz et al., 2010). The SLRE has warmer summer temperatures and higher productivity and calcium concentrations than Lake Superior (Loken et al., 2016), explaining the ability of *Dreissena* to establish and persist there. We know less about *Dreissena* establishment potential in APIS and along the south shore (at mouths of rivers), where productivity is only slightly elevated above levels of open Lake Superior, and Ca^++^ concentrations are on the upper end of the limit of uncertainty in veliger survivability (Lafrancois et al., 2018).

A 2017 APIS survey was implemented because of concerns over reported adult *Dreissena* on shipwrecks and native mussels by the National Park Service.A small number of *D. polymorpha* colonies (<20 adults) were found within and near APIS from 2015–2017 (Lafrancois et al., 2019). The goal of the survey was to determine *Dreissena* prevalence and distribution and the baseline and potential impacts on zooplankton and benthic communities (Trebitz et al., 2019). No settled juvenile or adult *Dreissena* were detected on passive samplers when retrieved (rock bags, Hester Dendy plates, cinder block anchors, lines, and buoys). *Dreissena* veligers were present in 43 zooplankton samples (44% of tows) but at low densities. Only 6/43 samples had densities greater than 5 specimens per m^2^. *Dreissena* DNA was found on 6 mesh substrates (16.2%).

One of the interesting findings was a spatial pattern from east to west in the islands, with greater densities in the west and north side of the islands and primarily absent from southeast islands (Trebitz et al., 2019). This pattern suggests that veligers could be transported from SLRE (largest, most established population in Lake Superior) to APIS (~100 km away) *via* summer currents (Beletsky et al., 1999, 2017; Bennington et al., 2010). *Dreissena* may be able to complete the veliger life stages over the three weeks to transport from SLRE to APIS, and many of these individuals would be predated on in the process, decreasing their abundance along the gradient from SLRE to APIS (Ackerman et al., 1994). Alternatively, there are coastal habitats and wetlands along the south shore of Lake Superior that may act as sources of veligers and other zooplankton species, although a much smaller scope than the SLRE.

The 2017 survey used genus-level identification, yet eDNA methods have expanded to identify *Dreissena* at the species level to determine whether zebra (more suitable for SLRE habitat) or quagga (more suitable for APIS habitat) dominate. Although eDNA methods are robust for aquatic invasive species detection, there may still be uncertainties in whether detection represents species presence or establishment (Sepulveda et al., 2020). Therefore, multiple lines of evidence, including multiple gear types and paired morphological and molecular datasets, are needed to assess *Dreissena* populations at low abundances.

We hypothesized that *Dreissena* veligers are transported by currents at low densities along the south shore of Lake Superior from the SLRE to the APIS. Specifically, we predicted that (1) *D. polymorpha* will dominate *Dreissena* detections because it is more suited to the estuary habitat. Additionally, with increased distance from the SLRE: (2) *Dreissena* DNA detection will decline in eDNA water and passive samples, with detection dropping off as density is very low; and (3) veliger density will decline; (4) “SLRE”-type zooplankton density will decline as no more individuals are locally added to the population and natural mortality due to predation and low resources reduces the population size; (5) veliger size will increase as they are growing during transport and no small veligers are locally added to the population. (6) Water quality (Ca^++^ and temperature) will shift from the estuary to the open lake consistent with mixing the warmer, Ca^++^ rich waters in the estuary to colder, Ca^++^ poor waters in the open lake.

## MATERIALS AND METHODS

### Survey Description

**FIGURE 1 |:**
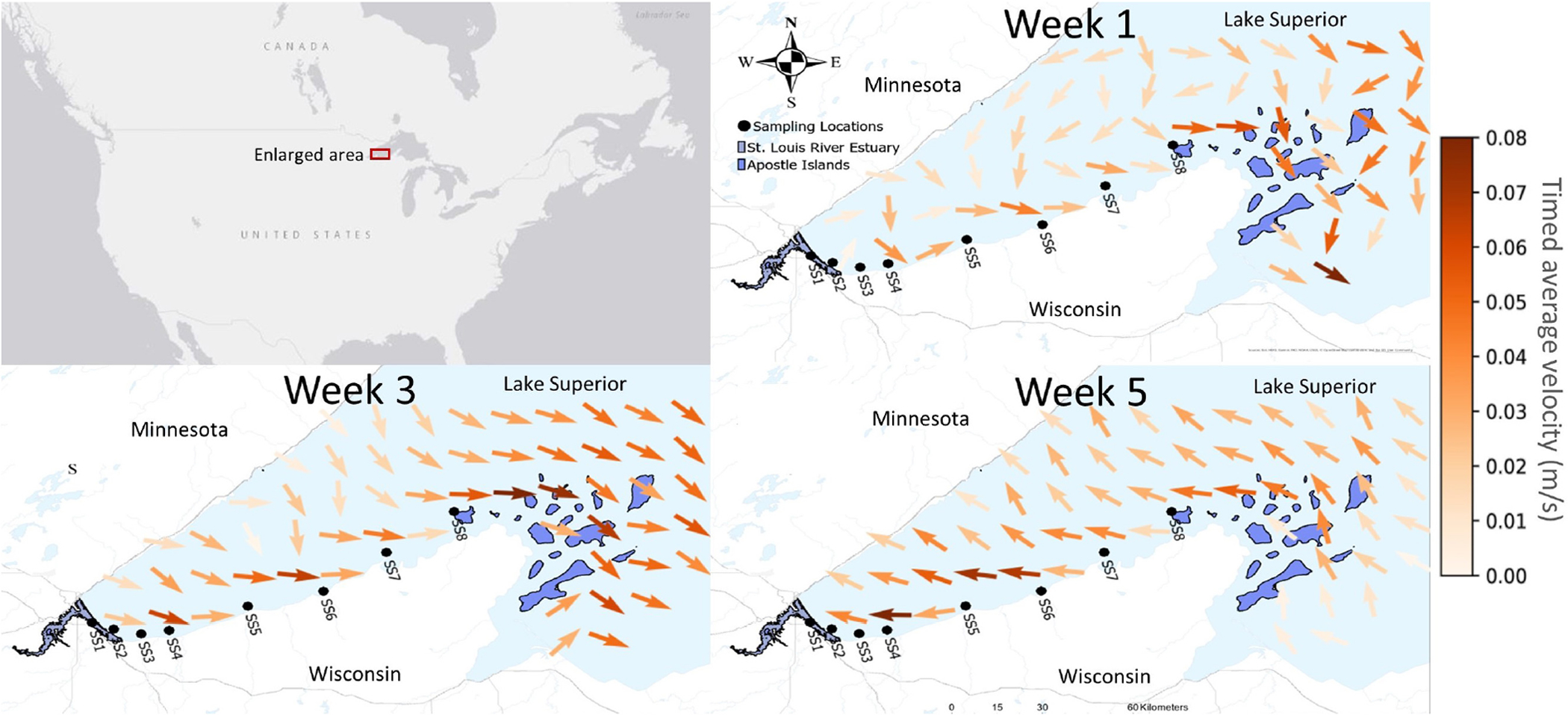
Maps displaying sampling sites and surface current patterns over the three sampling periods, with legend at right (in meters per second). Timed average velocity for two weeks prior to sampling (obtained from Great Lakes Coastal Forecasting System) were used to generalize the current patterns of the zooplankton community exiting the St. Louis River estuary (SLRE) would experience. Currents in week 1 and week 3 sampling trended eastward from the SLRE to the Apostle Islands (APIS), while they trended westward in the two weeks prior to Week 5.

In this study, eight sites (depth of 10–15 m) from the SLRE to the APIS were surveyed for *Dreissena*, zooplankton community, and environmental parameters (Figure 1 and Table 1). The four sites closest to the SLRE were grouped ~7 km apart with the first site in the Superior Bay area of the SLRE serving as a positive control (known *Dreissena* population). The four furthest east sites along the south shore to Sand Island, APIS were grouped ~17.5 km apart. There were three sampling time points over summer 2019: August 12–14th (week 1), August 29 (week 3), and September 16–17 (week 5). This collection coincided with peak veliger dispersal in the Great Lakes (Bowen et al., 2018).

### Environmental Datasets

Surface current patterns (time-averaged velocity) were determined by averaging the surface current velocity and direction for 2 weeks prior to the last sampling day of a sampling trip using data from the Great Lakes Coastal Forecasting System (GLCFS) database. Two-week averages were used because that is the estimated transportation time from the SLRE to APIS along longshore surface currents, determined by a glider deployed concurrently with the first sampling week that measured surface current velocity from the SLRE to APIS (Austin, 2013). The GLCFS forecasts numerous environmental variables for the Great Lakes based on stationary information derived from observation buoys throughout the Great Lakes forecasted in hourly increments. One dimensional depth averaged forecasted output datasets including time, latitude, longitude, east surface water velocity, and north surface water velocity at 10 km cells in the western arm of Lake Superior were downloaded as NetCDF files and plotted.

Additional environmental parameters were obtained during each sampling station visit. Temperature, conductivity, fluorescence, corrected photosynthetically active radiation (PAR), depth, pH, specific conductance, and dissolved oxygen concentration measurements were obtained *via* a SeaBird profile that was post-processed to bin values into 1 m depth intervals. Because calcium (Ca^++^) is thought to be a limiting factor for *Dreissena* shell formation, its concentration was measured *via* atomic absorption spectrophotometry in a filtered (0.45 μm hydrophilic nylon filter) and acidified (pH < 4) water sample obtained by combining near-surface and near-bottom water collected with Niskin bottles (Hoffman et al., 2010).

### Field Sample Collection and Processing

Three sampling methods were used to obtain *Dreissena* DNA: mesh substrate, water samples, and zooplankton tows. Mesh substrates were deployed on August 12–14 (week 1) and retrieved September 16–17 (week 5). Banners were constructed using a 1.2 m × 1.2 m 4′×4′ section of high-density polyethylene (HDPE) construction fencing. The 1.2 m × 1.2 m section was folded in half to create a 2-ply, 0.6 m wide × 1.2 m long banner. Each banner was secured to a mooring line through the center of the banner with ties at the bottom, center, and top. Two banners were secured per line [bottom (1 m from lake bottom) and surface (1 m from lake surface)] and attached to two buoys and concrete anchor. One buoy was positioned 1/2 to 1 m below the surface of the water for stability in minimizing drag during flow events, and another buoy was positioned at the surface to mark the location and assist with retrieval. Retrieved banners were placed into separate large ziplock bags (1 banner/bag) and stored at −80°C until processing. Each frozen mesh substrate was scraped on each side with a clean rubber squeegee to remove contents, rinsed, and filtered through a 63 μm sieve. Contents were preserved with 95% ethanol.

Water samples (1 L) were collected at the bottom (2 m above benthos) and surface (3 m below surface) using a certified clean HDPE collection bottle. Additionally, 1 field control was collected per day of sampling by filling a sample bottle with sterile water on the back deck of the boat where sample water was collected. eDNA water samples were filtered immediately after collection in the research vessel laboratory. Prior to sampling, the ship’s lab was thoroughly cleaned (all surfaces wiped with bleach solution), and the lab space was isolated from the surrounding area by taping heavy plastic sheeting from floor to ceiling to prevent contamination. To filter, water samples and controls were poured into sterile, single-use 250 ml Nalgene filter cups and filtered through a 1.2 μm polycarbonate filter membrane. Sample water was poured into the same filter cup until 1 L of water was filtered. If a filter clogged, an additional filter was used to finish filtering the sample. Two lab controls (before and after filtering all samples) were obtained each filtering day by filtering 250 ml of sterile water. Filters from samples and controls were put in sterile 2 ml microcentrifuge tubes and stored at −80°C until they could be vacuum desiccated.

*Dreissena* veligers and the zooplankton community were sampled by vertical net (0.5 m diameter, 64 μm mesh) at each site. Four vertical tows (2 m from bottom to surface) were composited into 1 sample. Afterward, each tow net contents were rinsed into a collection cup (a bucket rinsed with site water) and then the composite sample was transferred to a clean storage bottle and preserved in 70% ethanol. After at least 24 h, ethanol from the initial field preservation was decanted into HDPE Nalgene sample jars for subsequent DNA analysis.

### DNA Extraction and Analysis

Mesh substrate contents, water sample filters, and decanted ethanol were extracted for DNA and analyzed with qPCR using *Dreissena* species-specific markers to determine if zebra, quagga, or both species were present in each sample because veligers cannot be identified to species using morphology. Ethanol used to preserve mesh substrate contents was evaporated and DNA from samples collected at sites 2–8 was extracted using (Qiagen DNeasy Blood and Tissue Kit, Valencia, CA, United States) per manufacturer’s instructions. Due to the much larger biomass collected in the SLRE control site, ethanol preservative was filtered and extracted with the same methods used for water sample filters (see below). Although DNA results can be variable based on filtration and extraction methods as demonstrated by Eichmiller et al. (2016), polycarbonate filters and (Qiagen kits, Valencia, CA, United States) used in this study optimize invertebrate DNA yield and are common practice in the field (Piggott, 2016; Hinlo et al., 2017).

Using the (Qiagen DNeasy PowerWater kit, Valencia, CA, United States), DNA was extracted from filtered water samples with the manufacturer’s protocol modified by using a (Retsch Mixer Mill MM300, Haan, Germany) to beat the bead tubes. *Dreissena* genus-specific PCR assay was conducted on all samples, and species-specific assays (*D. polymorpha* and *D. bugensis*) were conducted for only those samples that were positive for *Dreissena* in the genus-specific PCR assay (at least 4 out of 8 replicates had measurable Ct values and the average of those Ct values were less than or equal to 40). The genus-specific qPCR used 16S primers developed by Gingera et al. (2017) (forward primer: TGGGGCAGTAAGAAGAAAAAAATAA, reverse primer: CATCGAGGTCGCAAACCG; probe: CCGTAGGGATAACAGC). Zebra mussel-specific COI primers (forward primer: GTGGTTGAACCTTATAYCCTCCTTT; reverse primer: AAATTGATGACATCCAGCACG; and probe: CAGGSCCTGAATGTCCTATAACTCTAGA) and quagga mussel specific cytochrome B primers (forward primer: CATTTCCTTATACCGTTTATTCTATTAGTACTCCT; reverse primer: AGGAGCTGTTTCGTGAGAAATATCA; and probe: TAGGATTTCTTCACACTACCGGG) were applied to samples determined to be positive for *Dreissena* DNA. Tests were conducted to ensure species specificity for *D. polymorpha* and *D. bugensis* PCR primers as described in Supplementary Document 1 following established guidelines (Goldberg et al., 2016). PCR conditions for all qPCR assays were 10 μl (TaqMan Environmental Master Mix 2 Applied Biosystems, Foster City, CA, United States), 1 μl of each forward and reverse primer at 10 μM (except for quagga, forward primer 1.8 μl), 2 μl of the probe at 2.5 μM, 3 μl of DNA template, and sterile water to bring the total volume to 20 μl. Eight qPCR replicates were run for each sample with the Dreissenid general marker, and 4 qPCR replicates were run per sample with species-specific markers. qPCR was conducted on a (QuantStudio 6 Flex Real-Time PCR System Applied Biosystems, Foster City, CA, United States), and cycling conditions consisted of an initial denaturation for 10 min at 95°C then 45 cycles of 95°C for 15 s and 60°C for 1 min. The reaction was monitored for fluorescence, indicating the presence of *Dreissena*, *D. polymorpha*, or *D. bugensis* DNA in the sample. Inhibition tests were conducted using Applied Biosystems^®^ (TaqMan^®^ Exogenous Internal Positive Control Reagents, Foster City, CA, United States) on all controls and samples. All qPCR tests were negative for inhibition, except for one mesh substrate sample from the SLRE. Two bottom eDNA water samples collected during week 1 at sites 4 and 5 were positive for target DNA using the general *Dreissenid* markers, but no signal was detected using species-specific markers. For these two samples, DNA copy #/μL for species-specific markers was inferred based on samples with a similar number (4–6) of positive replicates and a mean Ct 39–40.

### Laboratory Processing

Zooplankton was enumerated using the EPA Great Lakes National Program Office method (Great Lakes National Program Office [GLNPO], 2016), with the important modification that veligers were also enumerated using the crustacean protocol (not the microzooplankton protocol), which greatly increases the probability of detecting low-density occurrences because a much larger proportion of the total sample is searched. The length was measured for the first 20 veligers encountered within each line attached to the Sedgewick-Rafter slides so that measurements were not skewed. For three samples, zooplankton ethanol qPCR analysis detected that *Dreissenid* DNA was present, yet no veligers were found in the sample using the GLNPO method which involves subsampling. Therefore, these positive DNA but negative veliger search samples were examined again, with the entire sample searched rather than a subset. In two of the three samples, veligers were found (and measured) using the whole sample search method and added to the counts in the zooplankton community dataset.

### Data Analysis

The Python programming language package “quiver” was used to determine direction and intensity from the east and north vectors supplied through the GLCFS NetCDFs. Individual time points were averaged over two weeks to get an estimate of two-week direction and intensity prior to the last sampling event of a sampling trip. The averaged vectors incorporating direction and velocity were plotted through the python package matplotlib. Plots were generated to get a general understanding of the velocity and direction of currents prior to each sampling trip.

Means and SE were determined for DNA copy numbers for *D. polymorpha* and *D. bugensis* in each gear method. Surface and bottom samples were pooled because there was no difference in the DNA quantities among the different habitats. Trends over distance from SLRE were examined using linear modeling and visualized using ggplot in R (R Core Team, 2021).

Within the zooplankton community, veliger volumetric density (number per m^3^) means and standard errors were determined for each sampling site to determine trends as the distance from the SLRE increased. Additionally, to determine if veligers were traveling along the surface currents similarly to estuarine zooplankton taxa several representative taxa that typically inhabit estuarine but not offshore waters of Lake Superior were identified. We did so by eliminating from consideration any zooplankton taxa in our dataset that also had regular occurrences in the offshore-focused 2006–2016 Lake Superior Cooperative Science and Monitoring Initiative surveys (Pawlowski and Sierszen, 2020), and from the taxa that remained, choosing three from diverse phylogenetic groups for visualization: *Daphnia retrocurva*, *Diaphanosoma birgei*, and *Mesocyclops* copepodites. Densities of these taxa were plotted alongside densities for *Dreissena* veligers, and Pearson’s correlation tests (considered significant at *p* < 0.05) were conducted to quantify the association between the estuarine zooplankton taxa and veligers. The association is a measure of how closely the veligers mimic the dilution pattern of estuarine zooplankton with distance from the SLRE and is potentially diagnostic of local sources or sinks along the south shore. Veliger lengths for each sampling time point were compared over distance from SLRE to determine trends in growth and compared to growth-limiting environmental parameters calcium and temperature.

## RESULTS

### The Physical and Chemical Environment

During the two weeks prior to sampling, currents in week 1 and week 3 trended eastward from the SLRE to APIS, whereas they trended westward during week 5 (Figure 1). Nearshore current velocities ranged from roughly 0.02 to 0.8 m/s during the sampling period, which equates to 1.7 to 6.9 km/d. The long-shore distance between the westernmost and easternmost sampling station is about 90 km, so under steady-flow conditions, current-driven transit times between the two range from roughly 13 to 52 days.

**FIGURE 2 |:**
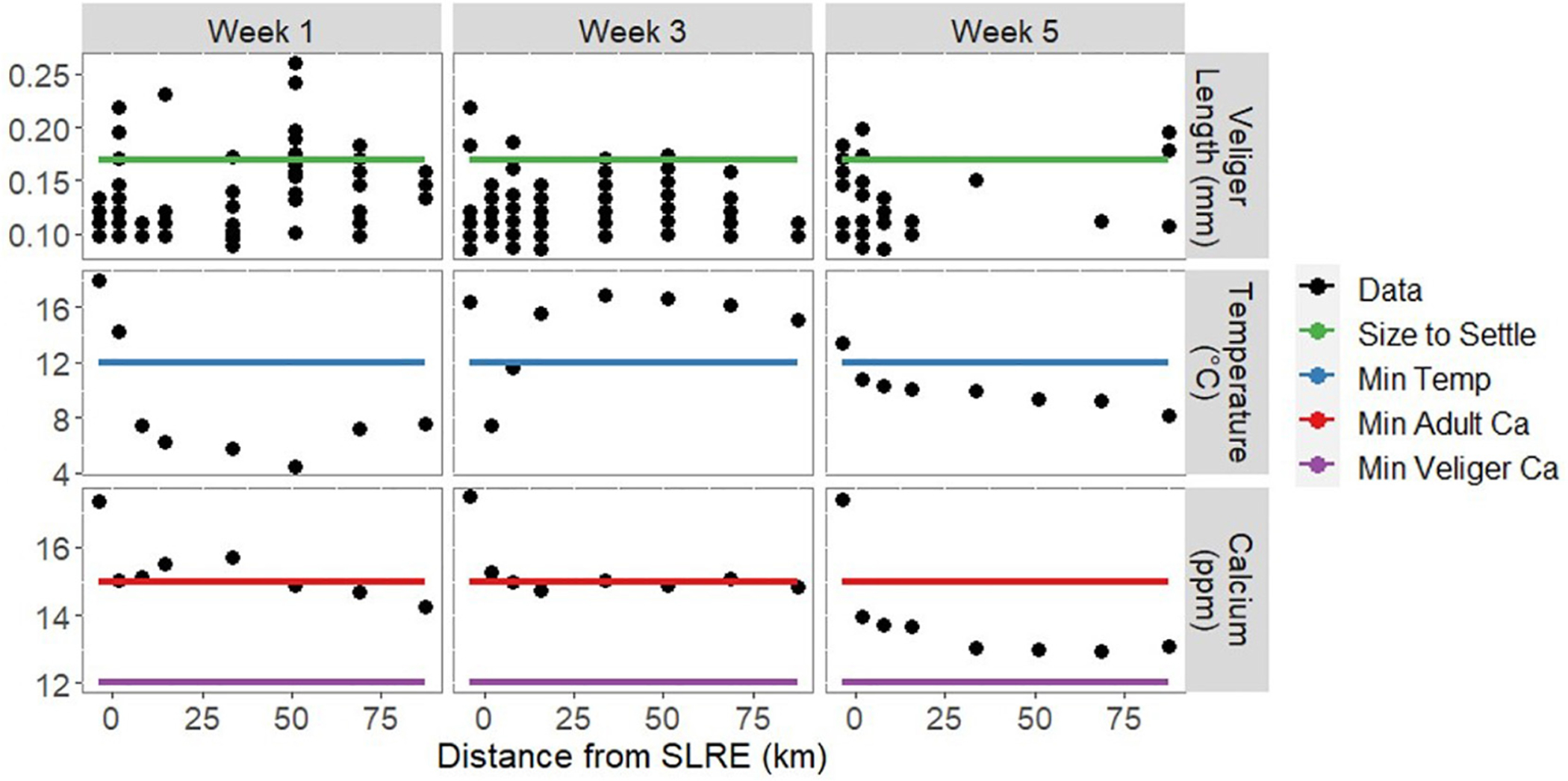
*Dreissena* veliger length (up to 20 measured individuals), mean water temperature, and Ca^++^ concentration over the three sampling weeks across the spatial gradient from the SLRE to APIS. Black points represent data obtained during this survey, and colored lines represent standard values for *Dreissena* development recorded in previous literature. *Dreissena polymorpha* veligers settle to grow as adults at 0.17 mm (green, Martel et al., 1994). *D. polymorpha* veligers require a temperature range of 12–24°C to develop (blue, McMahon, 1996). *Dreissena* spp. Require Ca^++^ concentrations greater than 12 and 15 ppm as veligers (purple) and adults (red), respectively (McMahon, 1996; Davis et al., 2015). We do not see a trend in size along the gradient from the SLRE to APIS, and some veligers collected during each sampling week met the size threshold for settling to the benthos to begin adult development. During each sampling week, multiple sites did not meet the temperature and calcium thresholds for veliger and adult development, respectively, but all sites had greater than the calcium concentrations necessary for veliger development.

The temperature averaged 11.0 (± 0.1) °C and ranged from 4.3–20.3°C (Figure 2). In weeks 1 and 5 temperature generally decreased along the south shore distance gradient, as would be expected with the mixing of water from the warmer estuary to the cooler lake. The highest temperature in week 3 was also recorded in the estuary, but there was not a consistent decrease along the shore. Calcium concentration (Ca^++^) averaged 14.8 (± 0.3) ppm and ranged from 13–17.5 ppm. During all sampling weeks, Ca^++^ was highest in the estuary and decreased as distance from the SLRE increased. Ca^++^ levels were lower in week 5 than in weeks 1 and 3 at all stations except the one inside the SLRE.

### Dreissena DNA

#### Mesh Substrates

**FIGURE 3 |:**
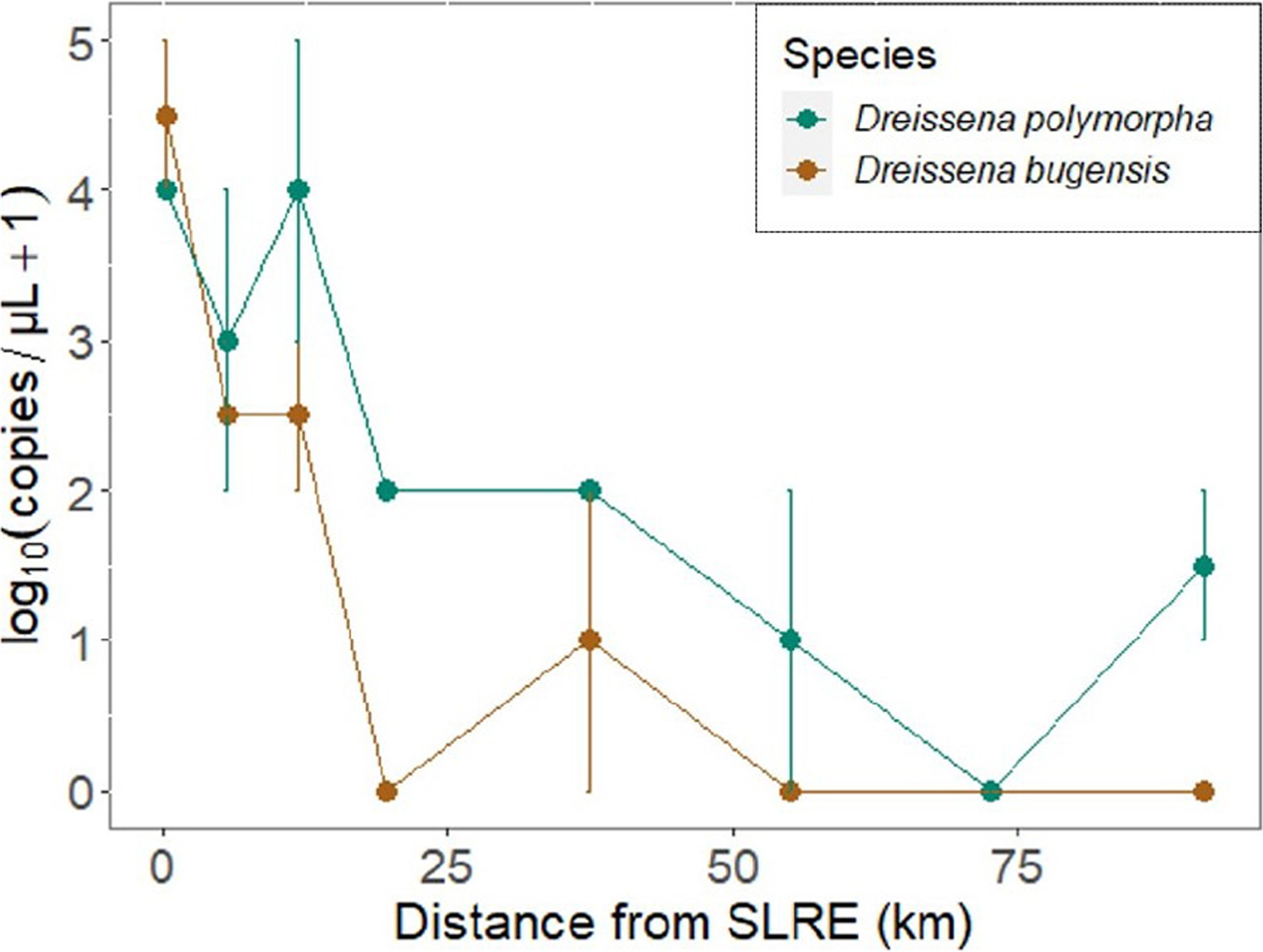
Mean DNA sequence abundance [log_10_(copies/μL + 1) ± SEM] of the quagga mussel (*Dreissena bugensis*: brown) and zebra mussel (*D. polymorpha*: blue) determined *via* qPCR from passive (mesh substrate) samples across the spatial gradient from the SLRE to APIS (in km). The distance 0 station is inside the SLRE. Surface and bottom samples had similar abundances and were pooled to determine means and SEs. *D. polymorpha* DNA abundance was greater than or equal to *D. bugensis* in all but one sample, and DNA copy numbers decreased as the distance from the SLRE increased.

*Dreissena polymorpha* DNA was detected in the ethanol preservative of 87.5% of mesh substrate samples, and *D. bugensis* DNA was detected in the ethanol from 50% of mesh substrate samples (Figure 3). For every 1 km increase in distance from the SLRE, mesh substrate DNA copy numbers decreased on average 8 and 9% for *D. polymorpha* and *D. bugensis*, respectively. *D. polymorpha* abundance was greater than or equal to *D. bugensis* in all but one sample, and *D. polymorpha* was on average 8 (± 2) times more abundant than *D. bugensis*.

#### Water Samples

**FIGURE 4 |:**
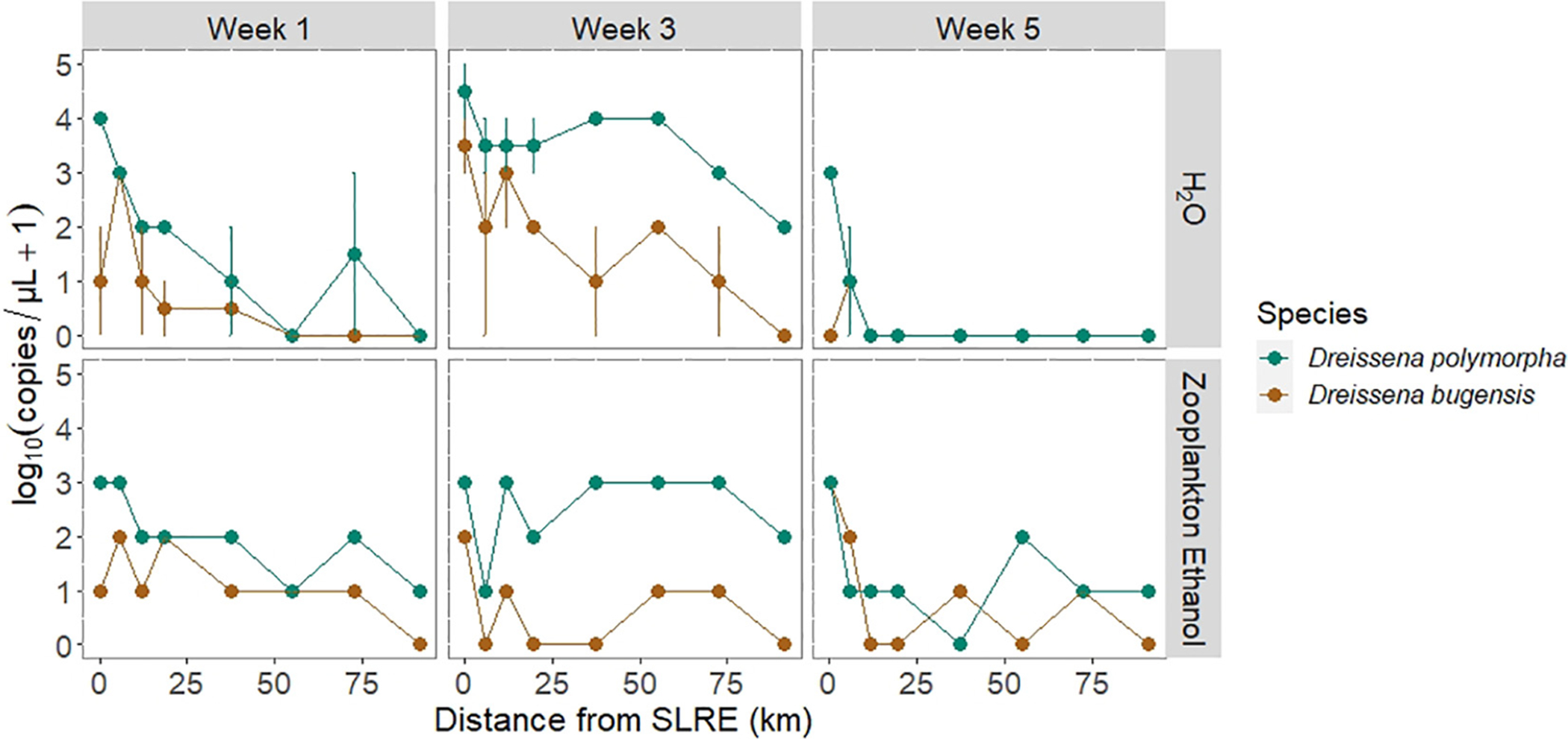
DNA sequence abundance [log_10_(copies/μL + 1)] of the quagga mussel (*D. bugensis*: brown) and zebra mussel (*D. polymorpha*: blue) determined *via* qPCR from water (H_2_O, mean ± SEM) and zooplankton ethanol samples over the three sampling weeks across the spatial gradient from the SLRE to APIS in km. Surface and bottom samples of H_2_O eDNA had similar abundances and were pooled to determine means and standard errors. *D. polymorpha* abundance was greater than or equal to *D. bugensis* in all but two samples, and detections decreased as the distance from the SLRE increased. Detections were very low in week 5 water eDNA samples when surface currents switched westward toward the SLRE.

*Dreissena polymorpha* and *D. bugensis* eDNA as detected in 62.5 and 37.5% of water samples, respectively (Figure 4). For every 1 km increase in distance from the SLRE, water sample eDNA copy numbers decreased on average 5 and 4% for *D. polymorpha* and *D. bugensis*, respectively. *D. polymorpha* abundance was greater than or equal to *D. bugensis* in all water samples, and *D. polymorpha* was on average 11 (± 1) times more abundant than *D. bugensis*. The lowest detections were found in week 5, with only 12.5% of samples detecting *D. polymorpha* or *D. bugensis* eDNA, compared to 62.5 and 100% in weeks 1 and 2, respectively.

#### Zooplankton Ethanol

*Dreissena polymorpha* and *D. bugensis* DNA as detected in 96 and 67% of zooplankton samples, respectively (Figure 4). For every 1 km increase in distance from the SLRE, DNA copy numbers determined from the ethanol in which zooplankton were preserved decreased on average 2 and 3% for *D. polymorpha* and *D. bugensis*, respectively. *D. polymorpha* abundance was greater than or equal to *D. bugensis* in all but two ethanol DNA samples, and *D. polymorpha* was on average 10 (± 1) times more abundant than *D. bugensis*. The lowest detections occurred in week 5 (September), with 3 (± 1) times greater copy numbers per μL in weeks 1 and 3 compared to week 5.

### Zooplankton Community

**FIGURE 5 |:**
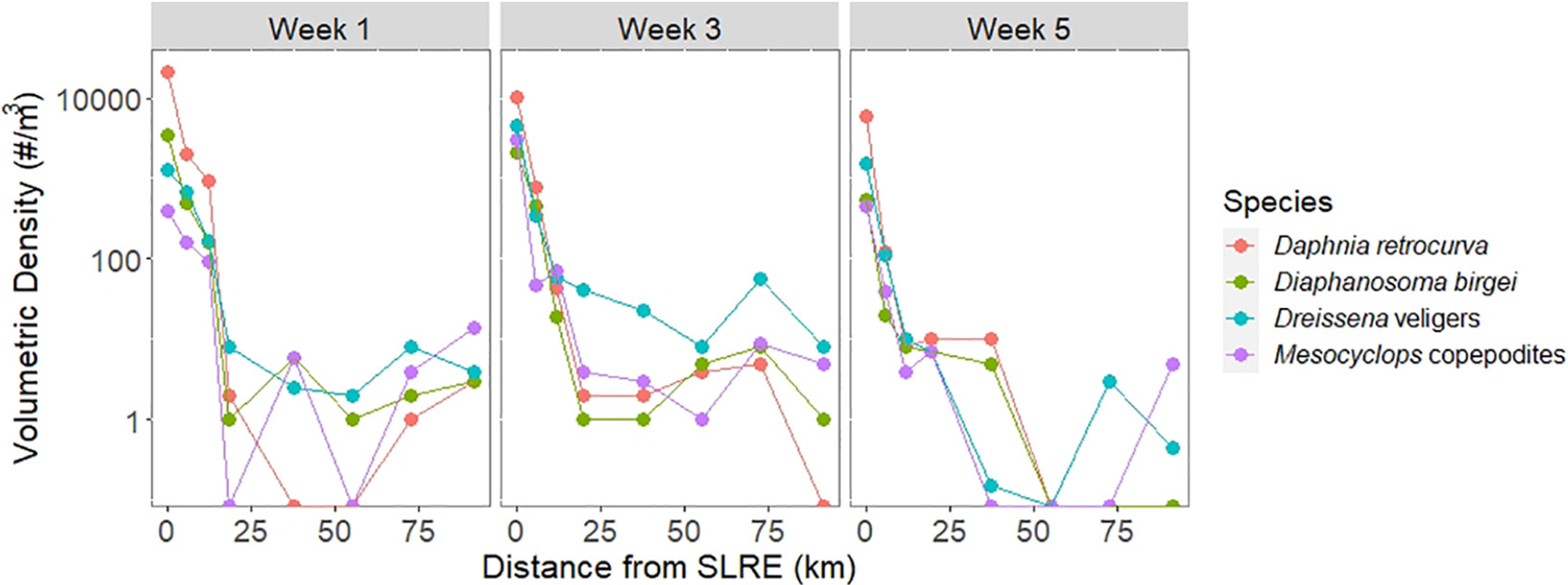
Volumetric density (log scale, number per m^3^) for *Dreissena* veligers (blue) and three zooplankton taxa representative of the estuary community: *Daphnia retrocurva* (red), *Diaphanosoma birgei* (green), and *Mesocyclops copepodites* (purple) in zooplankton tow samples over the three sampling weeks across the spatial gradient from the SLRE to APIS (km). The abundance for all four species decreased as the distance from the SLRE increased during all sampling weeks. The three zooplankton species correlated strongly (Pearson’s *r* > 0.9) with *Dreissena* veligers density.

*Dreissena* veligers (*N* = 304) were measured for length, averaging 122 (± 2) μm and ranging from 85–261 μm (Figure 2). There was no trend of increasing or decreasing size as the distance from SLRE increased. *Dreissena* veligers were present in all morphologically enumerated samples except for one sample collected in week 5 at the distance of 55 km from SLRE (Figure 5). For every 1 km increase in distance from the SLRE, veliger volumetric density (number per m^3^) decreased on average 5% (assuming a linear trend), although that decrease happened most quickly in the first 20 km and more slowly thereafter. There was no discernable difference in the rate of decrease or overall volumetric density of *Dreissena* veligers between the three sampling weeks.

Veliger trends over distance from the SLRE were compared to three phylogenetically diverse zooplankton taxa that are found in littoral areas but not open Lake Superior waters as an additional indicator of potential transport and showed a very similar longshore pattern (Figure 5). *Daphnia retrocurva* volumetric density decreased on average 7% and significantly correlated to *Dreissena* veliger volumetric density (*r* = 0.92, *p* < 0.01, *N* = 24). *Diaphanosoma birgei* decreased on average 5% and significantly correlated to *Dreissena* veligers (*r* = 0.91, *p* < 0.01, *N* = 24). *Mesocyclops* copepodites decreased on average 4% and significantly correlated to *Dreissena* veligers (*r* = 0.91, *p* < 0.01, *N* = 24).

## DISCUSSION

### Dreissena Detection

The goal of this study was to use molecular, morphological, and environmental evidence to determine if conditions were consistent with *Dreissena* veligers being out-washed from the SLRE and transported to a distant archipelago (APIS) *via* longshore currents. All *Dreissena* detection methods found a log-scale decrease in abundance from the SLRE to APIS, supporting this hypothesis. Except for the sampling stations near the SLRE, the veliger densities determined from zooplankton sample enumeration were very low, less than 10 individuals per m^3^. This is very different from the other four Great Lakes (Lakes Erie, Huron, Michigan, and Ontario) where *Dreissena* veliger densities across the summer reproductive season range from 10,000 to >100,000 individuals per m^3^ (Bowen et al., 2018; Burlakova et al., 2018) and further supports that the veligers found in nearshore Lake Superior are out-washed propagules being depleted by dilution and predation rather than being replenished from local production. One exception to this trend was the mesh substrate *D. polymorpha* abundance, which increased slightly at the site closest to APIS. This may be because it is close to a Sand Island local colony of *D. polymorpha*. The sharply declining gradient from west to east sampling stations was consistent among all sampling techniques, demonstrating the robustness of eDNA methods in detecting *Dreissena* even at low abundances. The highest abundances were detected in mesh substrate and water samples, compared to zooplankton ethanol samples; therefore, these methods may be most useful in future molecular detection surveys for *Dreissena*. Future studies may increase replication over space along the south shore to allow for occupancy modeling of PCR replicates, which would further characterize the sensitivity of the PCR assay.

*Dreissena polymorpha*, which is estuarine adapted (Karatayev et al., 2015), was more abundant than the open-lake adapted *D. bugensis* in samples collected along the south shore of Lake Superior, further supporting our hypothesis of an estuarine out-wash. But there was more *D. bugensis* collected in samples than expected, especially at the site within the SLRE. In mesh substrate samples, *D. polymorpha* and *D. bugensis* DNA quantities (copy numbers) were similar at the SLRE station, although DNA copy numbers were about an order of magnitude lower for *D. bugensis* than *D polymorpha* at the other sampling stations. DNA copy numbers at the SLRE station were also similar between the two species for some (but not all) of the weeks in the zooplankton ethanol and eDNA water data. This pattern was unexpected because recent formal benthos surveys from the SLRE –a ~600 sample effort by the University of Minnesota–Duluth’s Natural Resources Research Institute in 2013 through 2015 (Dr. Valerie Brady, personal communication), a ~40-sample effort by EPA in 2015 (unpublished data), and a ~200-sample effort by the Minnesota Pollution Control Agency in 2018 through 2020 (Dr. Kurt Schmude, personal communication)–have found only *D. polymorpha* among specimens large enough to fully identify. However, this does not rule out smaller individuals being *D. bugensis* (Angradi et al., 2017). We know that *D. bugensis*, first found in the SLRE in 2005 (Grigorovich et al., 2008), remains present as we have found it in the occasional benthic sled sample, and development and testing of the qPCR marker we are using included *D. bugensis* specimens sourced from 2012 United States Fish and Wildlife bottom trawls in the SLRE survey.

It is possible that *D. bugensis* in the SLRE release more veligers per adult than the numerically much more abundant *D. polymorpha*; plausible since *D. bugensis* can outcompete *D. polymorpha* in other ways such as surviving on fewer food particles, being active at lower temperatures, and having stronger substrate attachment (Karatayev et al., 2015; Ginn et al., 2018; Hetherington et al., 2019; D’Hont et al., 2021). Interlake cargo ships visiting the SLRE also might release ballast water containing veligers that are predominantly *D. bugensis* because that is the dominant species of *Dreissena* in lower Great Lakes locations (Karatayev et al., 2021). However, there is no prior data on veliger species composition, because, until the development of the species-specific *Dreissena* qPCR markers that we use here, researchers were unable to determine which *Dreissena* species veligers were from. A final possibility is that *D. polymorpha* and *D. bugensis* hybridize in the SLRE (Voroshilova et al., 2010), in which case the qPCR marker would identify the veligers as *D. bugensis* rather than *D. polymorpha* because it targets a portion of the mitochondrial DNA that is maternally inherited. Reports from the National Park Service indicate that *Dreissena* mussels in APIS look slightly more like *D. bugensis* and were found on soft substrates (*D. bugensis* habitat), but they were identified as *D. polymorpha* based on genetics (Dr. Brenda Lafrancois, personal communication). Further sequencing of *Dreissena* samples across the south shore would be a useful tool in establishing the source of these veligers.

Another interesting aspect of *Dreissena* detection was the false negatives and false positives detected *via* morphological and molecular analysis of zooplankton tows. Three samples were positive for *Dreissena* DNA, yet no veligers were found in the initial zooplankton search (conducted on a subsample). When these samples underwent a more thorough search, two of three contained veligers. Therefore, two samples were false negatives morphologically. The other sample (DNA positive, veliger negative) probably represents the lesser detection capability of morphological identification at low *Dreissena* abundances, given *Dreissena* DNA was detected at that site during other time points (Darling et al., 2021). These false negatives demonstrate the importance of using multiple detection methods at low abundances, which is especially important given detection impediments to DNA used in management (Darling and Mahon, 2011; Trebitz et al., 2017).

### Current Transport

Current variability influenced *Dreissena* abundance along the south shore of Lake Superior during our survey. During weeks 1 and 3, lake currents moved in the normal west to the east direction (Beletsky et al., 1999, 2017; Bennington et al., 2010), but in week 5 this was reversed. *Dreissena* in water samples had a consistent gradient from the SLRE to APIS in weeks 1 and 3, but no such gradient in week 5, presumably connected to these changes in currents. Although the general trend of Lake Superior currents is from the west to the east direction along the south shore, there is natural variability due to water temperature, air temperature, and wind patterns (Sun et al., 2020; Austin and Elmer, 2021). This variability is expected to increase due to climate change (Lam and Schertzer, 1999). The implication of this veliger transport system is if currents change direction and environmental conditions align, veligers may be transported elsewhere in Lake Superior. This has happened in other marine systems, including the case of the invasive jellyfish *Phyllorhiza punctata*, which spread globally through medusae drift in water currents. Abnormal current patterns introduced them to Malta and Italy (Deidun et al., 2017), the Gulf of Mexico (Johnson et al., 2005), and coastal Georgia (Verity et al., 2011). Veliger monitoring is necessary for other parts of Lake Superior, as currents may transport veligers to other areas in the lake.

These currents transport other estuarine zooplankton species. Zooplankton species are normally found in Lake Superior estuaries but not the open lake, such as *Daphnia retrocurva*, *Diaphanosoma birgei*, and *Mesocyclops* copepodites exhibited similar patterns of log-scale decline across the south shore. Similar to our findings, when *P. punctata* jellyfish were found in the Gulf of Mexico for the first time, large mats of *Sargassum* seaweed spp. were also reported washing up on beaches (Graham et al., 2003). Because these seaweed mats are found along with the loop current that *P. punctata* medusae normally drift in, it suggests an abnormal current coming off this loop that transported both the *Sargassum* and *P. punctata* to the Gulf of Mexico. Using multi-species and community-wide trends provides important evidence of co-transport along currents.

The alternative to the current-driven transport of veligers originating from the SLRE is that *Dreissena* veligers were sourced locally in our samples, i.e., from hitherto unknown bottom-settled *Dreissena* occupying the Lake Superior south shore or transported from coastal habitat and tributaries separate from the SLRE. If this was the case, we would not have seen a difference in our detections when the currents changed direction. Instead, in water samples, there was a decrease in detection during week 5 when currents changed direction. Although we do not have direct evidence of the sourcing of these veligers, the patterns described in this study as a whole suggest current transport of veligers originating from the known SLRE population is likely.

### Veliger Growth

We expected to see veliger size increase along the south shore gradient as veligers grew and developed over time along the transport route, but size did not increase. This may be evidence of local veliger sourcing or that veligers are settling out of the transported population. *D. polymorpha* veligers settle to grow as adults at 170 μm (Martel et al., 1994). Veligers collected during each sampling week met the size threshold for settling in the benthos to begin adult development, so this could impact the size distribution. It also suggests that if environmental and habitat conditions aligned, veligers collected at south-shore sites intermediate to SLRE and APIS could form the basis for additional settled adult *Dreissena* colonies in the future. However, the continued growth of the remaining smaller veligers should also cause the lower end of the size distribution to shift upward along the transport path, which we did not see evidence for.

We may not have seen veliger growth because there were not enough resources to grow, and the environmental conditions were not optimal. *D. polymorpha* veligers require a temperature range of 12–24°C to develop (McMahon, 1996). This threshold was not met in south shore sites beyond the SLRE in weeks 1 and 5. Additionally, *Dreissena* spp. require Ca^++^ concentrations greater than 12 and 15 ppm as veligers and adults, respectively (McMahon, 1996; Davis et al., 2015). Although all samples met the 12 ppm Ca^++^ threshold for veliger growth, only those at or close to the SLRE met the adult threshold consistently. But the temperature and Ca^++^ measurements were very close to the threshold, and with environmental change, may allow for veliger development and settlement in the future. As with the current patterns, variation in environmental parameters may allow a “window of invasiveness” for *Dreissena* in Lake Superior to new suitable habitats. This underscores the importance of early detection monitoring to locate and respond to any such new incursions.

## CONCLUSION

Current patterns are an important vector for biological invasions in marine systems and represent an understudied component of species spread in the Great Lakes. Through DNA, morphological, and environmental surveys along the south shore of Lake Superior, we demonstrated veliger transport from an invaded estuary (SLRE) to a distance archipelago (APIS) by nearshore currents. These currents and environmental conditions can be variable, and increasingly so with climate change; therefore, early detection monitoring for *Dreissena* is important for continued conservation of Lake Superior biodiversity and habitat. Veligers can disperse by currents across a hostile, food-limited, and chemically unfriendly island and establish in modestly favorable habitat, allowing for a “window of invasiveness,” adding to the ecological understanding of *Dreissenid* invasion risk. Future studies will investigate the species composition of the SLRE *Dreissena* population and investigate the current transportation of *Dreissena* from the SLRE to other known colonies in Lake Superior. We look forward to the additional species-level information on *Dreissena* veligers that the new qPCR markers make possible, which will add granularity to the understanding of veliger distribution patterns around the Great Lakes and in other aquatic systems where this highly invasive mollusk genus is spreading.

## Supplementary Material

Supplement1

## Figures and Tables

**TABLE 1 |:** Summary of survey methods.

Gear	Method	Analysis intent	Sample size
GLFS	Currents inferred from two-week average prior to sampling	Match current trends to *Dreissena* and other zooplankton detection	3
Calcium water sample	Bottom and surface combined	Cation analysis method	24
CTD sonde	Vertical profile with 0.25 m depth bins for temperature (C) and other water chemistry parameters.	Match values to *Dreissena* development stages	24
Mesh substrate	2, 0.6 × 1.2 m mesh substrates sampled at bottom (2 m above benthos) and surface (2 m below surface).	qPCR targeting *Dreissena* (markers for genera and species specific: *D. polymorpha* and *D. bugensis*).	8 bottom and 8 surface
eDNA water sample	1L water collected at bottom (2 m above benthos) and surface (3 m below surface)	qPCR targeting *Dreissena* (markers for genera and species specific: *D. polymorpha* and *D. bugensis*).	24 bottom, 24 surface, and 6 controls
Zooplankton tow	Composite four vertical tows per sample starting 2 m from bottom. Decanted ethanol from preserved samples used for DNA analysis.	qPCR targeting *Dreissena* (markers for genera and species specific: *D. polymorpha* and *D. bugensis*), morphological enumeration for veliger densities, zooplankton community.	24

The eight stations were sampled three times over 5 weeks; mesh substrates were deployed over 5 weeks and retrieved at the end of the study period. The Great Lakes Forecasting System (GLFS) dataset was obtained from the NOAA database and not generated from the survey.

## Data Availability

The datasets presented in this study can be found in online repositories. The names of the repository/repositories and accession number(s) can be found below: https://edg.epa.gov/.
