# Supplementary material for: Invasive Dreissena Mussel Coastal Transport From an Already Invaded Estuary to a Nearby Archipelago Detected in DNA and Zooplankton Surveys
Source: Front Mar Sci. Author manuscript; Available in PMC 2023 Feb 21. (PMC9016628; doi:10.3389/fmars.2022.818738)
Supplement: Supplement1 [file NIHMS1796429-supplement-Supplement1.docx]

Supplementary Document 1

Primers for the two markers, cytochrome b (cytB) for quagga mussel and cytochrome oxidase subunit I (COI) for zebra mussel, were first developed by comparing available mitochondrial DNA sequence data for these species from GenBank in 2018. These sequences were aligned and compared with DNA sequence data for COI and cytB from various North American unionid mussel species. PCR primers and probes were then chosen to cover species-specific regions for the two *dreissenid* targets (Table S1). The cytB marker designed for quagga mussel detection targets the same loci as a similar marker published previously for zebra mussel (Gingera et al. 2017) but included nucleotide substitutions at key locations to allow for specificity to quagga mussel. Primers were then tested against synthetic gBlock sequences to maximize annealing temperatures and primer concentrations for PCR conditions to produce the lowest Ct values with the highest relative fluorescence units (RFU). Primers were then tested on the target *dreissenid* species DNA and against DNA samples of the other non-target *dreissenid* species and 13 different native unionid species from 9 genera (Table S2). Tissue vouchers from either the target species (zebra and/or quagga mussel) were obtained from sampling locations within the Great Lakes Basin and included sampling locations in the Upper Lakes including Lakes Erie, Huron and Superior. DNA was extracted and tested with qPCR using the zebra-mussel specific (DreCOI03b) and quagga mussel specific (QMcytBGing) was carried out to verify specificity. In each qPCR 1 nanogram of each DNA was added. The zebra mussel-specific qPCR primers and probe successfully amplified only samples from that species with no amplification of non-targets (Table S2). Conversely, the quagga mussel-specific cytB qPCR primers and probe successfully amplified only samples from that species with no amplification of non-targets.

Table S1. qPCR Primer and Probe Sequences for DreCOI03b (zebra-mussel specific) and QMcytBGing (quagga-mussel specific).

|  | Marker | Sequence (5’-3’) |
| --- | --- | --- |
| *DreCOI03b* | DreCOI03bF | GTGGTTGAACCTTATAYCCTCCTTT |
|  | DreCOI03bR | AAATTGATGACATCCAGCACG |
|  | DreCOI03bP | 6FAM- CAGGSCCTGAATGTCCTATAACTCTAGA –ZEN/Iowa Black |
| *QMcytBGing* | *QMcytBGingF* | CATTTCCTTATACCGTTTATTCTATTAGTACTCCT |
|  | *QMcytBGingR* | AGGAGCTGTTTCGTGAGAAATATCA |
|  | *QMcytBGingP* | VIC- TAGGATTTCTTCACACTACCGGG –MGB |

Table S2. Mussel species and specimens tested for qPCR specificity.

| Sample ID | Waterbody | State | Species | Common Name | Year Sampled | *Dre*  *COI03b* | *QM*  *cytB* |
| --- | --- | --- | --- | --- | --- | --- | --- |
| DP5842 | St. Louis River | MN/WI | *Dreissena polymorpha* | Zebra Mussel | 2015 | + | - |
| DP5832 | St. Louis River | MN/WI | *Dreissena polymorpha* | Zebra Mussel | 2015 | + | - |
| DP5837 | St. Louis River | MN/WI | *Dreissena polymorpha* | Zebra Mussel | 2015 | + | - |
| DP5834 | St. Louis River | MN/WI | *Dreissena polymorpha* | Zebra Mussel | 2015 | + | - |
| DP770936 | Lake Huron/Erie Corridor | MI | *Dreissena polymorpha* | Zebra Mussel | 2014 | + | - |
| DP771456 | Lake Huron/Erie Corridor | MI | *Dreissena polymorpha* | Zebra Mussel | 2014 | + | - |
| DP771416 | Lake Huron/Erie Corridor | MI | *Dreissena polymorpha* | Zebra Mussel | 2014 | + | - |
| DP771636 | Lake Huron/Erie Corridor | MI | *Dreissena polymorpha* | Zebra Mussel | 2014 | + | - |
| DB770996 | Lake Huron/Erie Corridor | MI | *Dreissena bugensis* | Quagga Mussel | 2014 | - | + |
| DB771116 | Lake Huron/Erie Corridor | MI | *Dreissena bugensis* | Quagga Mussel | 2014 | - | + |
| DB770956 | Lake Huron/Erie Corridor | MI | *Dreissena bugensis* | Quagga Mussel | 2014 | - | + |
| DB770776 | Lake Huron/Erie Corridor | MI | *Dreissena bugensis* | Quagga Mussel | 2014 | - | + |
| DB2789 | St. Louis River | MN/WI | *Dreissena bugensis* | Quagga Mussel | 2012 | - | + |
| DB2787 | St. Louis River | MN/WI | *Dreissena bugensis* | Quagga Mussel | 2012 | - | + |
| DB1_2779 | St. Louis River | MN/WI | *Dreissena bugensis* | Quagga Mussel | 2012 | - | + |
| DB2776 | St. Louis River | MN/WI | *Dreissena bugensis* | Quagga Mussel | 2012 | - | + |
| DB2_2779 | St. Louis River | MN/WI | *Dreissena bugensis* | Quagga Mussel | 2012 | - | + |
| DB15001 | St. Louis River | MN/WI | *Dreissena bugensis* | Quagga Mussel | 2015 | - | + |
| DB15002 | St. Louis River | MN/WI | *Dreissena bugensis* | Quagga Mussel | 2015 | - | + |
| DB15003 | St. Louis River | MN/WI | *Dreissena bugensis* | Quagga Mussel | 2015 | - | + |
| DB15004 | St. Louis River | MN/WI | *Dreissena bugensis* | Quagga Mussel | 2015 | - | + |
| DB15005 | St. Louis River | MN/WI | *Dreissena bugensis* | Quagga Mussel | 2015 | - | + |
| DB15006 | St. Louis River | MN/WI | *Dreissena bugensis* | Quagga Mussel | 2015 | - | + |
| B16-001 | Allegheny River | PA | *Ligumia recta* | Black Sandshell | 2016 | - | - |
| B16-003 | Allegheny River | PA | *Ligumia recta* | Black Sandshell | 2016 | - | - |
| B16-012 | Allegheny River | PA | *Strophitus undulatus* | Creeper | 2016 | - | - |
| B16-013 | Allegheny River | PA | *Strophitus undulatus* | Creeper | 2016 | - | - |
| B17-007 | Allegheny River | PA | *Elliptio complanata* | Eastern Elliptio | 2017 | - | - |
| B17-010 | Allegheny River | PA | *Elliptio complanata* | Eastern Elliptio | 2017 | - | - |
| B17-003 | Allegheny River | PA | *Lampsilis radiata* | Eastern Lampmussel | 2017 | - | - |
| B17-005 | Allegheny River | PA | *Lampsilis radiata* | Eastern Lampmussel | 2017 | - | - |
| B16-014 | Allegheny River | PA | *Lasmigona costata* | Fluted-shell | 2016 | - | - |
| B16-016 | Allegheny River | PA | *Lasmigona costata* | Fluted-shell | 2016 | - | - |
| B16-007 | Allegheny River | PA | *Ptychobranchus fasciolaris* | Kidneyshell | 2016 | - | - |
| B16-009 | Allegheny River | PA | *Ptychobranchus fasciolaris* | Kidneyshell | 2016 | - | - |
| B16-023 | Allegheny River | PA | *Ortmanniana ligamentina* | Mucket | 2016 | - | - |
| B16-025 | Allegheny River | PA | *Ortmanniana ligamentina* | Mucket | 2016 | - | - |
| B17-001 | Allegheny River | PA | *Potamilus alatus* | Pink Heelsplitter | 2017 | - | - |
| B17-015 | Allegheny River | PA | *Potamilus alatus* | Pink Heelsplitter | 2017 | - | - |
| B16-010 | Allegheny River | PA | *Lampsilis cardium* | Plain Pocketbook | 2016 | - | - |
| B16-011 | Allegheny River | PA | *Lampsilis cardium* | Plain Pocketbook | 2016 | - | - |
| B16-020 | Allegheny River | PA | *Lampsilis ovata* | Pocketbook | 2016 | - | - |
| B16-022 | Allegheny River | PA | *Lampsilis ovata* | Pocketbook | 2016 | - | - |
| B16-017 | Allegheny River | PA | *Villosa iris* | Rainbow | 2016 | - | - |
| B16-019 | Allegheny River | PA | *Villosa iris* | Rainbow | 2016 | - | - |
| B16-004 | Allegheny River | PA | *Elliptio dilatata* | Spike | 2016 | - | - |
| B16-006 | Allegheny River | PA | *Elliptio dilatata* | Spike | 2016 | - | - |
| B16-026 | Allegheny River | PA | *Lampsilis fasciola* | Wavy-rayed Lampmussel | 2016 | - | - |
| B16-028 | Allegheny River | PA | *Lampsilis fasciola* | Wavy-rayed Lampmussel | 2016 | - | - |
